# Percutaneous Coronary Intervention for Iatrogenic Right Coronary Artery Dissection Post Bentall Procedure: A Case Report and Minireview

**DOI:** 10.1155/2018/3420721

**Published:** 2018-10-29

**Authors:** Sameer Saleem, Mubbasher A. Syed, Khalid Changal, Abdulelah Nuqali, Mujeeb Sheikh

**Affiliations:** ^1^Presence Saint Joseph Hospital, Chicago, IL 60657, USA; ^2^Division of Cardiovascular Medicine, University of Toledo Medical Center, Toledo, OH 43614, USA; ^3^Mercy St. Vincent Medical Center, Toledo, OH 43608, USA; ^4^Internal Medicine, George Washington University, 2121 I St NW, Washington, DC 20052, USA

## Abstract

Iatrogenic coronary artery dissection is a potentially life-threatening complication of cardiovascular interventions. The optimal management of iatrogenic coronary artery dissection is not clear; however, both conservative management and percutaneous or surgical revascularization have been performed depending on the patient's clinical status and the extent of dissection. We present the first reported case of right coronary artery dissection after Bentall procedure performed for ascending aortic aneurysm. Urgent percutaneous intervention using adjunctive coronary imaging was performed with excellent clinical recovery. In this article, we highlight coronary artery dissection after Bentall procedure as a possible complication, provide an insight into various options in its management, and review published data on iatrogenic coronary artery dissection. We also discuss the challenges in percutaneous treatment of coronary artery dissection with special focus on intracoronary imaging for accurate diagnosis and guidance in the management of this complex lesion.

## 1. Case Presentation

**Figure 1:**
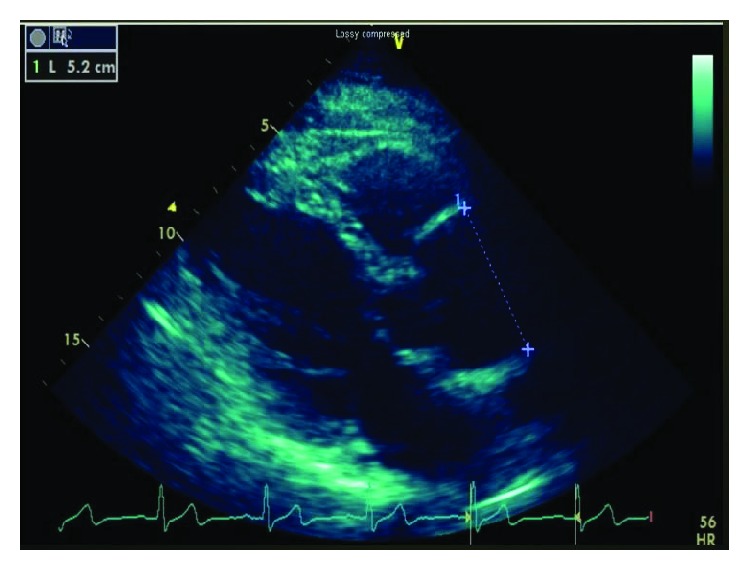
A parasternal long axis view on transthoracic echocardiography demonstrating an enlarging aortic root at 5.2 cm.

We present a 66-year-old Caucasian male with a history of hypertension and chronic type A aortic dissection who was found to have an enlarging aortic root measuring 5.2 cm in diameter on an echocardiography done as part of surveillance of aortic dissection repair done 9 years ago using a tube graft with resuspension of the aortic valve (Figure 1). Echocardiography was followed by CT aortography that showed the aortic root measuring 6.0 cm × 5.4 cm in diameter. The patient denied any symptoms and an elective surgical reconstruction was planned. Preoperative coronary angiogram showed normal coronary arteries.

**Figure 2:**
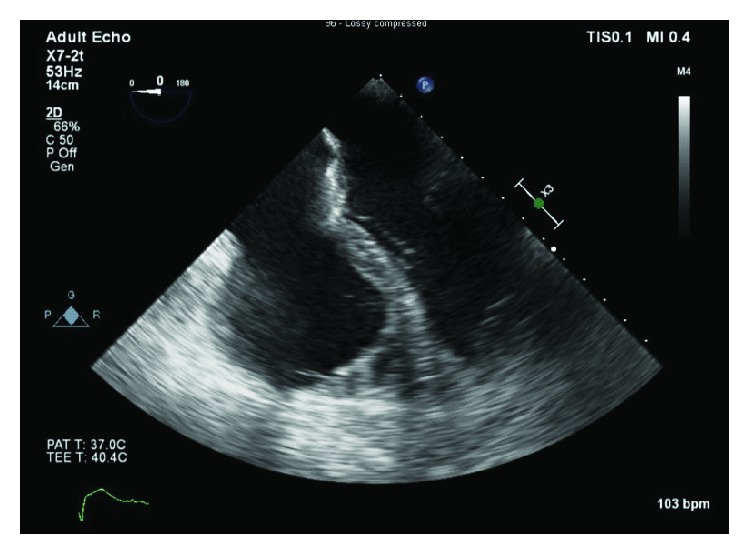
Transesophageal echocardiography post cardiac arrest revealed severe right ventricular dilatation.

The patient subsequently underwent modified Bentall procedure. This involved graft replacement of the aortic root, replacement of the aortic valve using a 27 mm bioprosthesis (St. Jude Medical Trifecta aortic bioprosthesis; St. Jude Medical Inc., St. Paul, MN, USA), and reimplantation of coronary arteries into the graft using the button technique. Soon after sternotomy closure was done, he was found in cardiac arrest with ventricular fibrillation intractable to pharmacologic resuscitation and defibrillation. Mediastinal reexploration was immediately performed revealing a fibrillating heart with no evidence of obvious bleeding or injury. He was internally defibrillated, and normal sinus rhythm was achieved. The patient was systemically heparinized and stabilized with vasopressors and veno-arterial extra-corporeal membrane oxygenation (VA-ECMO). Urgent transesophageal echocardiography (TEE) was done that showed a new, severe dilatation of the right ventricle along with reduced ejection fraction but a normal left ventricle (Figure 2). The prosthetic aortic valve was intact.

**Figure 3:**
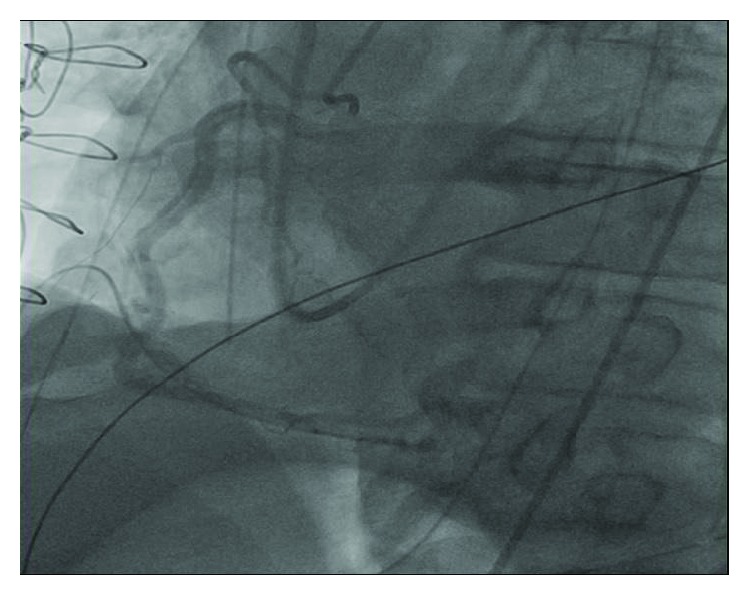
Catheterization showing a spiral dissection extending from the ostium of the right coronary artery (RCA) down to its distal segment, sparing the bifurcation.

**Figure 4:**
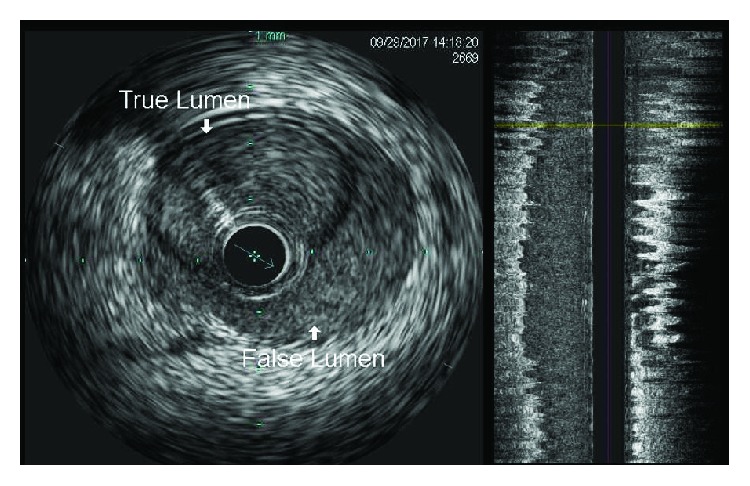
Intravascular ultrasound (IVUS) demonstrating the true and false lumens of coronary artery dissection.

**Figure 5:**
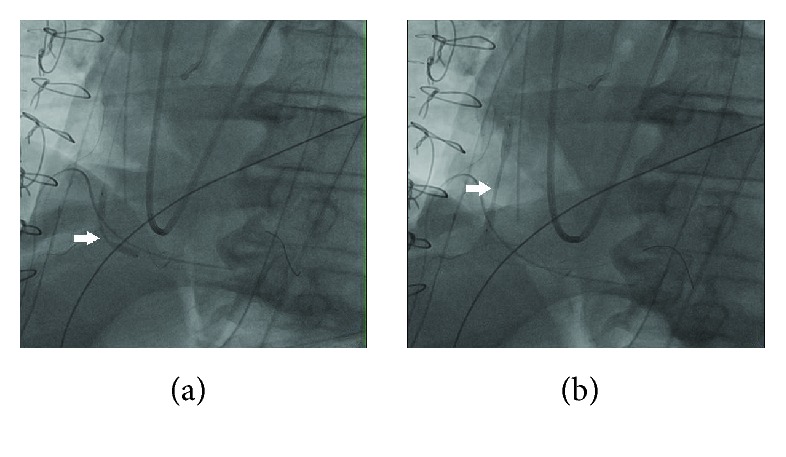
Angiogram showing stent deployment in the right coronary artery at the site of dissection as marked by white arrows in panels (a) and (b).

**Figure 6:**
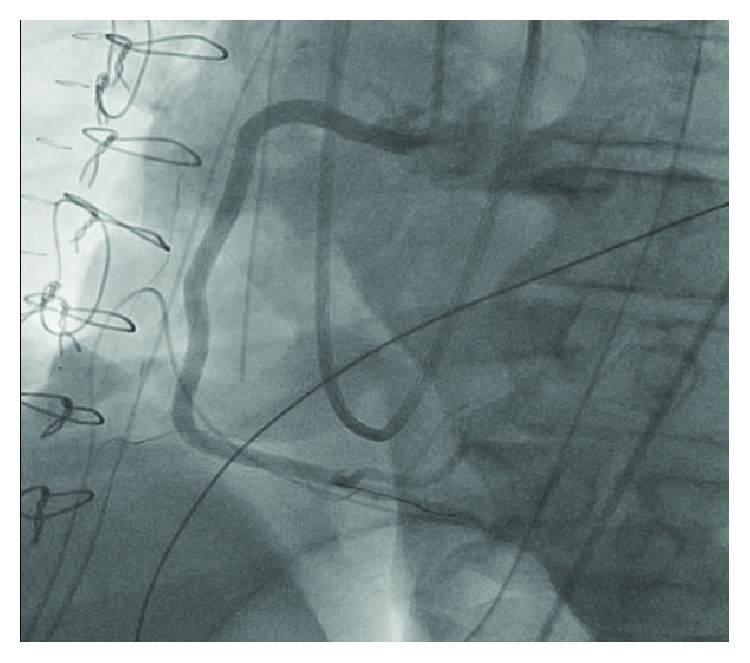
Restoration of TIMI-3 blood flow in the posterior descending and posterolateral branches of the right coronary artery (RCA) following placement of multiple stents in the RCA.

A concern for iatrogenic injury to the coronary vessels prompted an emergent coronary angiography which revealed dissection of the right coronary artery (RCA) extending from the ostium down to its distal segment, sparing the bifurcation (Figure 3; see Video 1 in Supplementary Materials). The left coronary artery was normal (see Video 2 in Supplementary Materials). A 6-French JR4 guiding catheter was placed in the ostium of the right coronary artery, and the dissection plane was traversed antegradely with a workhorse coronary guidewire (Prowater). Intravascular ultrasound (IVUS) using the Volcano Eagle Eye IVUS catheter was performed to ensure location of the wire in the true lumen distally, evaluate the nature and extent of coronary dissection, and select appropriate size of coronary artery stents. IVUS confirmed a spiral coronary dissection spanning between the distal RCA and ostium and sparing the right posterolateral and right posterior descending arteries (Figure 4). A 3.5 × 32 mm drug eluting stent (SYNERGY, Boston Scientific) was placed in the distal segment followed by four 4.0 mm drug eluting stents (SYNERGY, Boston Scientific) in overlapping fashion under IVUS guidance that restored TIMI-3 blood flow in the posterior descending and posterolateral branches of the right coronary artery (Figures 5(a), 5(b), and 6; see Videos 3–8 in Supplementary Materials).

**Figure 7:**
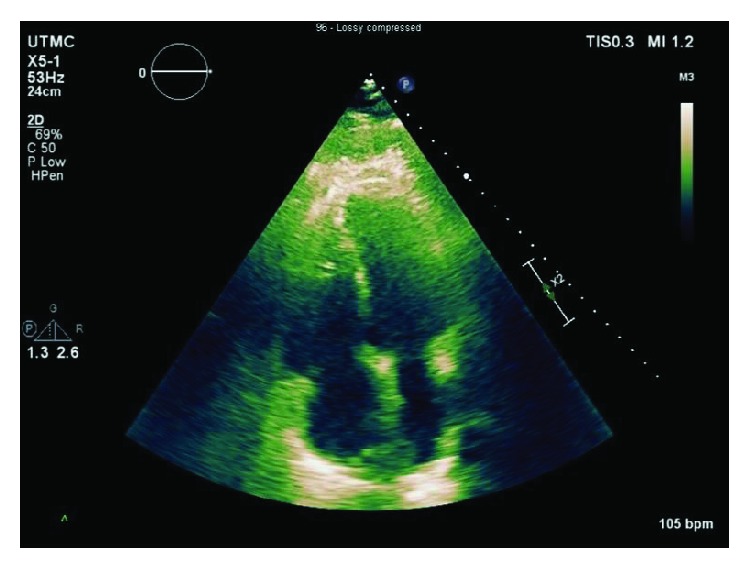
Transthoracic echocardiography one week later showing improvement in right ventricular size and function.

His postoperative course was also complicated by a right-sided pneumothorax which required chest tube drainage and nonoliguric renal failure that were managed conservatively. The patient's clinical status showed marked improvement, and by the fourth day postsurgery, he was extubated and weaned off from hemodynamic support. A transthoracic echocardiography one week later showed improved right ventricular size and function (Figure 7). The patient was subsequently transferred to rehab hospital where he had an uneventful six weeks of cardiac rehabilitation. He was subsequently discharged home in a stable condition. He has been doing well on follow-up.

## 2. Discussion

The Bentall procedure for management of aortic root dilatation was first described in 1968 by Bentall and De Bono. This involves surgical replacement of the ascending aorta and aortic valve with composite tubular graft containing a prosthetic valve followed by reimplantation of coronary arteries into holes punched in the graft [1]. The Bentall procedure has remained the procedure of choice for aortic root replacement with a reported survival of 91.7% at 10 years [2]. The classical Bentall procedure carries a risk of postoperative hemorrhage and pseudoaneurysm formation [3, 4]. Over the years, improvements and modifications have been proposed to minimize complications [5–7]. Button reimplantation that involves removal of a full-thickness “button” of the aorta surrounding the coronary ostia and implanting them into the openings made in the vascular graft is one technique that has yielded excellent results [6, 8]. The disadvantages of this technique include longer time required for dissecting out the coronary ostia, especially the ostium of the left coronary artery, and the risk of damage or occlusion of the left main coronary artery, the circumflex artery, or the first septal perforator branch [6]. Moreover, with this technique, mobilization of the coronary ostia is difficult and may require additional sutures to the left coronary ostial anastomosis to obtain hemostasis [6].

Reports regarding coronary artery dissection after the Bentall procedure are lacking in literature. We describe the first reported case of RCA dissection after the Bentall procedure. We think that coronary artery dissection in our case was probably related to either mechanical injury during excision and reimplantation of the coronary buttons or from instrumentation at the time of administration of cardioplegics into the coronary arteries [9].

We performed literature review in PubMed using the search strategy “Iatrogenic[Title] AND Coronary[Title] AND Dissection[Title].” This yielded 82 articles; out of which, 38 articles had detailed information on the type of management strategy for each patient with iatrogenic coronary artery dissection and thus were selected for review. Four of these articles were case series studies, and the rest were case reports. We identified a total of 48 patients who suffered iatrogenic coronary artery dissection (ICAD), 30 (62.5%) of which were females. The age group with the most number of patients reported (i.e., 29.2%) was 61–70 years. 47 patients developed ICAD as a result of coronary angiography whereas only one patient developed ICAD after a coronary artery bypass graft (CABG) procedure. We did not find any case of ICAD resulting from the Bentall procedure. 41 patients had some form of intervention either with stent placement (33 patients), CABG (6 patients), or both (2 patients) whereas only 6 patients had conservative treatment as part of the management of ICAD. One patient died before any management strategy could be instituted. Dissection occurred in the left coronary circulation in 32 (66.7%) and right coronary circulation in 16 (33.3%) patients. Intracoronary imaging with either intravascular ultrasound (IVUS) or optical coherence tomography (OCT) was employed in 15 patients for evaluation and guidance in management. These findings are summarized in Table 1. The details of the 38 selected articles are provided in tabulated form as a supplementary item with this review (see “Articles selected in systemic review” in Supplementary Materials).

The diagnosis of coronary artery dissection with angiography alone can be arduous since an angiogram is a two-dimensional luminogram and does not image the arterial wall that is affected in coronary dissection [10]. Thus, advanced imaging techniques in addition to angiography are needed for accurately diagnosing and guiding management [10]. Intravascular ultrasound (IVUS) and optical coherence tomography (OCT) are two such diagnostic modalities considered gold standard in accurately diagnosing coronary dissection [10–12]. IVUS allows a deeper and longer assessment of vessels thus enabling adequate visualization and better appreciation of the extent of intramural hematoma as compared to OCT [10, 13]. This is because of insufficient optical penetration and shadowing associated with OCT [13]. However, OCT has a higher spatial resolution and is superior to IVUS in detecting intimal tears, false lumen, and also intramural hematomas [13]. Amongst noninvasive imaging studies, multislice computed tomography (MSCT) is an attractive modality that can detect a double-barreled coronary lumen or low-density signal surrounding the lumen in an intraluminal hematoma; however, its utility is limited by low spatial resolution that may diagnose proximal but not the distal vessel involvement [14, 15].

The overall management of ICAD is often dependent on operator discretion and other clinical and patient variables including the severity of dissection and hemodynamic stability. A wide variety of management strategies that include conservative management, percutaneous intervention (PCI), emergent coronary artery bypass grafting, thrombolytic therapy, and cardiac transplantation have been done [16–19]. However, percutaneous approach is still the preferred and the quickest way to restore coronary flow and improve hemodynamics in cases of ongoing ischemia [16, 20–25]. Treatment of coronary artery dissection with percutaneous intervention is challenging. In one study, 28 (65%) amongst 43 patients who underwent PCI for spontaneous coronary artery dissection had technical success [26].

There are multiple reasons for suboptimal results associated with PCI in coronary artery dissection. First, with dissection, it is difficult to advance a guidewire into the true lumen [27]. Second, there is a risk of extension of the intramural hematoma in the forward or backward direction during the procedure that may further jeopardize coronary blood flow [27]. Moreover, depending on the location of the dissection, stent deployment may be a demanding task [27]. A dissection in a small vessel may not be amenable to stent placement whereas a larger dissection even if stented carries a risk of malapposition after the intramural hematoma has resorbed over time [27]. More often than not, the dissection can be extensive, requiring longer stents and thus increasing the risk of in-stent restenosis [27].

Some authors have recommended percutaneous intervention techniques to improve results in patients with coronary artery dissection. In case of a relatively focal lesion, selection of a longer stent that spans across both the proximal and distal edges of the lesion would be preferable [28]. This will prevent extension of the intramural hematoma [28]. In cases of longer lesions, deploying stent in the distal segment followed by the proximal area and then the middle segment of the dissection in order to prevent propagation of intramural hematoma has been advocated [28].

Intracoronary imaging modalities can be of great value in guiding successful PCI. IVUS and OCT can be used to identify true lumen, intimal tears and extent of the dissection, and intramural hematoma. They can ensure proper placement of guidewire in the true lumen and help in selecting the appropriate size of coronary stent and its optimal deployment [29, 30]. Intracoronary imaging can also verify adequate obliteration of the false lumen and compression of the intramural hematoma after the stent has been placed [29, 30]. Thus, in cases where there is suspicion of iatrogenic coronary artery dissection, we strongly recommend IVUS as an important adjunct to achieve high technical success rate while treating such complex lesions.

## 3. Conclusion

Iatrogenic right coronary artery dissection should be considered in patients with ventricular fibrillation and acute right ventricular dilatation after Bentall procedure. Percutaneous intervention along with intracoronary imaging is a useful strategy to accurately diagnose and guide revascularization in these cases.

## Figures and Tables

**Table 1:** Findings from literature review.

		Number of patients (%)
Total number of patients		48 (100%)
	Male	18 (37.5%)
	Female	30 (62.5%)

Age group (in years)	31–40	4 (8.3%)
	41–50	8 (16.7%)
	51–60	11 (22.9%)
	61–70	14 (29.2%)
	71–80	10 (20.8%)
	81–90	1 (2.1%)

Procedure complicating into ICAD	Coronary angiography	47 (97.9%)
	CABG	1 (2.1%)

Territory involving the ICAD	Right coronary circulation	16 (33.3%)
	Left coronary circulation	32 (66.7%)

Management strategy^∗^	Conservative management	6 (12.5%)
	Stent placement	33 (68.7%)
	CABG	6 (12.5%)
	Both stent and CABG	2 (4.2%)

Intracoronary imaging		15 (31.2%)
	IVUS	9 (18.7%)
	OCT	6 (12.5%)

Follow-up/in-hospital course	Stable	38 (79.2%)
	Death	2 (4.2%)
	Exertional angina	1 (2.1%)
	Aortic insufficiency	1 (2.1%)
	Unknown outcome	6 (12.5%)

CABG: coronary artery bypass graft; ICAD: iatrogenic coronary artery dissection; IVUS: intravascular ultrasound; OCT: optical coherence tomography. ^∗^1 patient died before any intervention was decided.

## Supplementary Materials

Supplementary Materials (1) Spiral right coronary artery dissection (Video 1). (2) Normal left coronary artery (Video 2). (3) First stent deployment (Video 3). (4) Second stent deployment (Video 4). (5) Third stent deployment (Video 5). (6) Fourth stent deployment (Video 6). (7) Final angiography showing Grade III TIMI flow (Video 7). (8) Final angiography showing Grade III TIMI flow (Video 8). (9) Articles selected in systemic review.
